# Cable Crosstalk Suppression with Two-Wire Voltage Feedback Method for Resistive Sensor Array

**DOI:** 10.3390/s16020253

**Published:** 2016-02-19

**Authors:** Jianfeng Wu, Shangshang He, Jianqing Li, Aiguo Song

**Affiliations:** School of Instrument Science and Engineering, Southeast University, Nanjing 210096, China; 220152718@seu.edu.cn (S.H.); ljq@seu.edu.cn (J.L.); a.g.song@seu.edu.cn (A.S.)

**Keywords:** networked resistive sensor array, measurement error, one-wire VF-NSDE circuit, two-wire VF-NSDE circuit

## Abstract

Using a long, flexible test cable connected with a one-wire voltage feedback circuit, a resistive tactile sensor in a shared row-column fashion exhibited flexibility in robotic operations but suffered from crosstalk caused by the connected cable due to its wire resistances and its contacted resistances. Firstly, we designed a new non-scanned driving-electrode (VF-NSDE) circuit using two wires for every row line and every column line to reduce the crosstalk caused by the connected cables in the circuit. Then, an equivalent resistance expression of the element being tested (EBT) for the two-wire VF-NSDE circuit was analytically derived. Following this, the one-wire VF-NSDE circuit and the two-wire VF-NSDE circuit were evaluated by simulation experiments. Finally, positive features of the proposed method were verified with the experiments of a two-wire VF-NSDE prototype circuit. The experiment results show that the two-wire VF-NSDE circuit can greatly reduce the crosstalk error caused by the cables in the 2-D networked resistive sensor array.

## 1. Introduction

Resistive sensor arrays are important for tactile sensing [1,2,3,4,5,6,7,8], infrared sensing [9,10], light sensing [11], *etc.* For limited space in sensitive areas, many resistive sensor arrays used in robotic applications are connected with their test circuits with different length cables. Fernando *et al.* [1] designed a 16 × 9 force sensor patch with its test cable’s length more than 55 mm to cover large distances within robots and machines for interacting with human beings. Speeter [2] reported a flexible sensing system with its test cable’s length more than 60 mm and achieved a sampling rate of more than 60 Hz in scanning 16 × 16 resistive taxels. Fernando *et al.* [3] realized and compared three circuits of networked piezoresistive sensor arrays with test cable length longer than 70 mm based on standard microcontrollers, programmable systems on chip, and field programmable gate arrays. Yang *et al.* [4] designed a 32 × 32 flexible array with a test cable length longer than 70 mm using PI-copper films within a 160 mm × 160 mm temperature and tactile sensing area, which could be used to recognize large objects of different shapes. Zhang *et al.* [5] reported a 3 × 3 thin tactile force sensor array with its test cable length longer than 95 mm based on conductive rubber. Castellanos-Ramos *et al.* [6] reported a 16 × 16 tactile sensor array with its test cable length greater than 100 mm based on conductive polymers with screen-printing technology. Kim *et al.* [7] reported a new concept of a flexible tactile sensor array with more length capable of sensing contact force and position with high performance and high spatial resolution. Lazzarini *et al.* [8] reported a 16 × 16 tactile sensor array for practical applications requiring manipulation with its test cable’s length of 500 mm. Saxena *et al.* designed a 16 × 16 bolometer array of IR detectors [9,10] and a 16 × 16 imaging array of light-dependent resistors [11]. With longer flexible test cables, the resistive sensor array modules are more flexible for robotic operations and easier to be connected with their test circuits.

In connected cables with fewer wires (*M* + *N* wires for a *M* × *N* resistive array), sensor modules of networked resistive arrays in shared row-column fashion were connected with their test circuits [11]. With equal length and the same material, wires of long cables had similar wire resistances which increased with the increase of cables’ length. Between the plugs of the connected cables and the sockets of the test circuits, there existed contacted resistances which varied in the range from 0 ohms to several ohms with the change of the contacted conditions caused by mechanical vibration and time variation. As for the voltage feedback circuits of the resistive networked sensor arrays in shared row-column fashion, the voltage differences between the circuits’ test ports and the sensor modules’ ports of the same connected wires were caused by the similar wire resistances and the different contacted resistances. Thus, the ideal feedback conditions in the voltage feedback circuits of the 2-D networked resistive sensor arrays were destroyed and crosstalk caused by the test cables existed. As such, good cable crosstalk suppression methods should be further studied.

For this purpose, we present a novel cable crosstalk suppression method with less cost for the 2-D networked resistive sensor arrays in the row-column fashion. This paper begins with an overview of the application fields of the 2-D networked resistive sensor arrays. Secondly, a novel cable crosstalk suppression method will be proposed and its equivalent resistance expression of the element being tested will be analytically derived. Followed, experiments will be implemented to evaluate this method with different parameters such as the wire resistances and contacted resistances of the cables, the array size, the measurement range of the EBT, and the adjacent elements’ resistances of 2-D networked resistive sensor arrays. Finally, the results of experiments will be analyzed and conclusions for the method will be given.

## 2. Principle Analysis

With the row-column fashion, few wires were use in the 2-D networked resistive circuits. However, these circuits suffered from the crosstalk for parasitic parallel paths in the 2-D networked resistive sensor arrays, and some theoretical analyses had been implemented for suppressing it. Fernando *et al.* [1,3,4,5,6,7,8,12,13,14] suppressed the crosstalk caused by the adjacent elements and the multiplexers with larger number of op-amps using virtual ground technique. With the Improved Isolated Drive Feedback Circuit (IIDFC) [15], Wu *et al.* depressed the crosstalk error caused by the adjacent column elements. With the Improved Isolated Drive Feedback Circuit with Compensation (IIDFCC) [16], Wu *et al.* reduced the influence on the EBT caused by parasitic parallel paths for the multiplexers’ channel resistor and the adjacent elements. In these methods, the measurement accuracy of the EBT still suffered from the interferences of cables’ parameters, such as wire resistances and contacted resistances. Liu *et al.* [17] defined the voltage feedback method including the voltage feedback non-scanned-electrode (VF-NSE) method, the voltage feedback non-scanned-sampling-electrode (VF-NSSE) method, and the voltage feedback non-scanned-driving-electrode (VF-NSDE) method. Wu *et al.* [18] proposed a general voltage feedback circuit model for the two-dimensional networked resistive sensor array.

**Figure 1:**
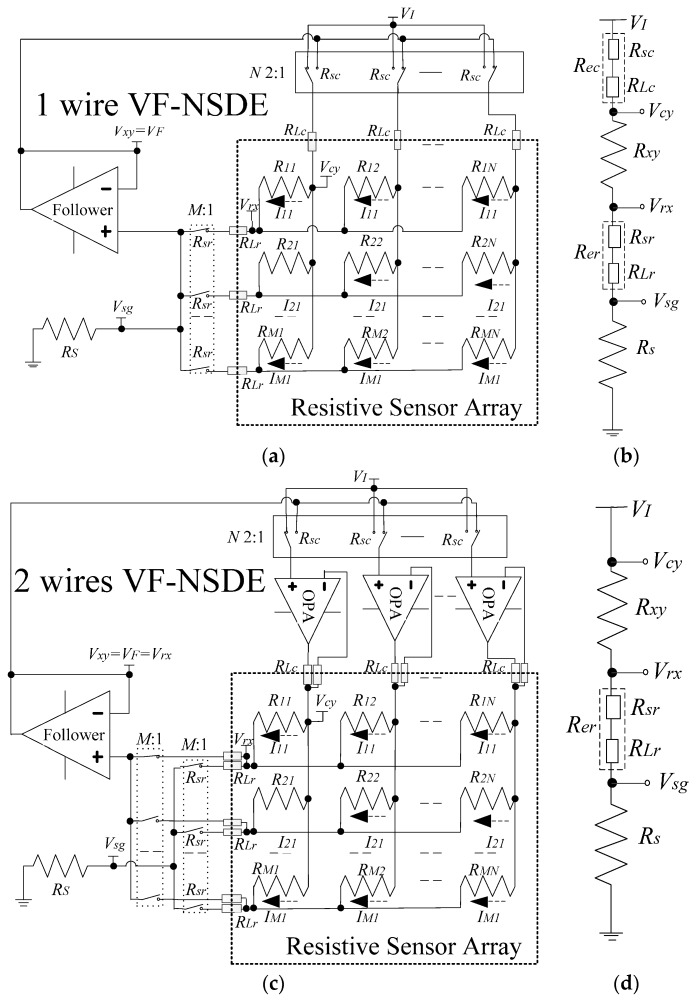
(**a**) One-wire VF-NSDE circuit (Circuit A); (**b**) simplified measurement circuit of a one-wire VF-NSDE circuit (Circuit B); (**c**) two-wire VF-NSDE circuit (Circuit C); and (**d**) simplified measurement circuit of a two-wire VF-NSDE circuit (Circuit D).

In this analysis, a VF-NSDE circuit was taken as the example. A traditional VF-NSDE circuit of a resistive networked sensor array in a shared row-column fashion is shown as Circuit A in Figure 1a. In Circuit A, *R_11_* in the *M* × *N* resistive array was the element being tested (EBT); only one connected wire was used for every row line and every column line between the array and the circuit; only one equal current *M*:1 multiplexer was used between the sampling resistor (*R_s_*) and the row lines of the sensor module. On the column lines of the circuit, 2:1 multiplexers had multiplexer switch resistances (*R_sc_*s); column wires had column resistances (*R_Lc_*s) including column wire resistances and contacted resistances. On row lines of the circuit, the equal current *M*:1 multiplexers had multiplexer switch resistances (*R_sr_*s); row wires had row resistances (*R_Lr_*s) including row wire resistances and contacted resistances. Thus, the Circuit A had one voltage feedback op-amp, *N* 2:1 multiplexers, and *M* + *N* wires.

Under an ideal condition, all *R_sc_*s and all *R_Lc_*s were omitted. Thus, the voltage (*V_cy_*) on the column line of EBT was equal to the set voltage (*V_I_*), and the voltages on non-scanned column lines were equal to the feedback voltage (*V_F_*). At the same time, all *R_sr_*s and all *R_Lr_*s were omitted. Thus, the voltage (*V_sg_*) on *R_s_* was equal to the voltage (*V_rx_*) on the row line of EBT. Under the effect of the ideal op-amp, *V_F_* followed the change of *V_sg_*. Thus, *V_F_*, *V_rx_*, and *V_sg_* were equal. As the voltages on non-scanned column lines were equal to *V_rx_*, the currents on the adjacent row elements of EBT were equal to zero. At the same time, the current on the non-inverting input of the ideal op-amp was omitted for its infinite input impedance, the current (*I_xy_*) on the EBT was equal to the current (*I_s_* = *V_sg_*/*R_s_* = (*V_I_* − *V_rx_*)/*R_xy_* = (*V_I_* − *V_sg_*)/*R_xy_*) on *R_s_*. As *V_I_* and *R_s_* were known, *V_sg_* = *V_F_* could be measured by ADC, so the equivalent resistance value (*R_xy_*) of the EBT in the Circuit A could be calculated with Equation (1):

(1) *R_xy_* = (*V_I_* − *V_F_*) × *R_s_*/*V_F_*

However, under real conditions, as shown in Figure 1b, *V_cy_* was not equal to *V_I_* for *R_sc_* and *R_Lc_*, and *V_sg_* was not equal to *V_rx_* for *R_sr_* and *R_Lr_*. The ideal feedback condition was destroyed by the row wires and the column wires, so extra measurement errors of the EBT occurred. For suppression crosstalk of the cables in the 2-D networked resistive arrays, we proposed a two-wire voltage feedback method as shown in Figure 1c.

In the Circuit C, we used two wires for every row line and every column line between the sensor module and the test circuit; also we used one column driving op-amp for every column line and one equipotential *M*:1 multiplexer between the row lines and the voltage feedback op-amp. Thus, Circuit C had one voltage feedback op-amp, *N* column driving op-amps, *N* 2:1 multiplexers, two *M*:1 multiplexers, and 2(*M* + *N*) wires.

Every column line in the sensor module was connected with the output of its column driving op-amp by one driving wire and it was also connected with the inverting input of its column driving op-amp by one driving sampling wire. The non-inverting input of every column driving op-amp was connected with the common port of its column 2:1 multiplexer, thus every non-inverting input was connected with *V_I_* or *V_F_*. The non-inverting input of the EBT’s column driving op-amp was connected with *V_I_* while the non-inverting inputs of other column driving op-amps were connected with *V_F_*.

As the input impedance of every column driving op-amp was much bigger than its *R_sc_*, the effect of all *R_sc_*s could be omitted. Thus, the voltage on the non-inverting input of its column driving op-amp was equal to the input voltage (*V_I_* or *V_F_*) on its 2:1 multiplexer. If the column driving op-amps had sufficient driving ability, the voltage on every column line followed the change of the voltage on the non-inverting input of its column driving op-amp. So *V_cy_* was equal to *V_I_*, and the voltages on non-scanned column lines were equal to *V_F_*. Thus the crosstalk effect of *R_Lc_*s and *R_sc_*s were suppressed.

By one equal current wire, every row line in the sensor module was connected with one channel of the equal current *M*:1 multiplexer with its common port connected with *R_s_*. In the equal current *M*:1 multiplexer, only the row line of EBT was gated and all other non-scanned lines were suspended. So, only the row line of the EBT was connected with *R_s_*.

By one equipotential wire, every row line in the sensor module was also connected with one channel of the equipotential *M*:1 multiplexer with its common port connected with the non-inverting input of the voltage feedback op-amp. In the equipotential *M*:1 multiplexer, only the row line of EBT was gated and all other non-scanned lines were suspended. Only the row line of the EBT was connected with the non-inverting input of the voltage feedback op-amp. From the EBT’s column driving op-amp, the test current firstly flowed through the EBT, then it flowed through the equal current wire, followed it flowed through the equal current *M*:1 multiplexer, finally it flowed through *R_s_* to ground.

As the input impedance of the voltage feedback op-amp was much larger than its series resistances including the switch-on resistance of the equipotential *M*:1 multiplexer, and the wire resistance and the contacted resistance of the equipotential, the voltage on the non-inverting input of the voltage feedback op-amp was equal to the voltage (*V_rx_*) of the EBT’s row line. Under the effect of the voltage feedback op-amp, *V_F_* followed the change of *V_rx_*. Thus, *V_F_* was equal to *V_rx_*. As the input impedance of the voltage feedback op-amp was much larger than its parallel resistances, such as *R_s,_ R_sr_*, and *R_Lr_*, the leak current on the non-inverting input of the voltage feedback op-amp could be omitted, as the voltage on every non-scanned column line was equal to *V_F_*, which was equal to *V_rx_*. Thus, the currents on the EBT’s (*N* − 1) row adjacent elements were zero. The current (*I_s_*) on *R_s_* was equal to the current (*I_xy_*) on the EBT. The current with equal value also flowed through *R_sr_* and *R_Lr_*. As *R_s_* was known and *I_s_* was equal to *I_xy_*, we know *I_xy_* if the voltage (*V_sg_*) on *R_s_* and the voltage (*V_xy_* = *V_cy_* − *V_rx_* = *V_I_* − *V_rx_*) on the EBT were known. Then we could get *R_xy_* of the EBT.

However, *V_sg_* was not equal to *V_F_* = *V_rx_* for *R_er_* (Figure 1d) which was the crosstalk caused by the row wire. Thus, extra measurement error of the EBT was created. From the above discussion, we could know that the currents on *R_xy_*, *R_s_*, and *R_er_* had equal values. We could use Equation (2) to calculate *R_xy_* in the Circuit C. We could find that no *R_er_* existed in Equation (2). As *V_I_* and *R_s_* were known, *V_sg_* and *V_F_* could be measured by ADC, so the equivalent resistance value (*R_xy_*) of the EBT in the Circuit C could be calculated with Equation (2). Thus, the crosstalk caused by the row wires was suppressed.

(2) *R_xy_* = (*V_I_* − *V_F_*) × *R_s_*/*V_sg_*

From the above discussion, the two-wire voltage feedback method can suppress the crosstalk caused by the row wires and the column wires, such as *R_sr_*s, *R_Lr_*s, *R_sc_*s, and *R_Lc_*s.

## 3. Experiments and Discussion

### 3.1. Simulation Experiments in NI Multisim

To emulate the performance of our method, a precise op-amp, OP07, was selected as the macro-model of the op-amp (from the datasheet, the offset voltage, the bias current, the gain-bandwidth, and the gain are equal to 10 μV, 0.3 nA, 0.6 MHz, and 112 dB, respectively) in the simulation experiments using National Instrument (NI) Multisim 12. In the simulation experiments, *V_I_* was 5.0 V.

#### 3.1.1. *R_0_* Effect Simulation Experiment in NI Multisim

**Figure 2:**
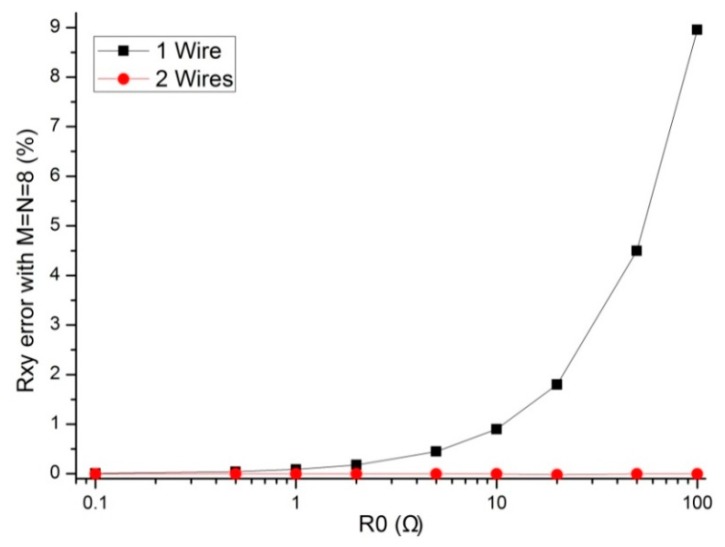
Effect of *R_0_* on the *R_xy_* errors in the one-wire VF-NSDE circuit and the two-wire VF-NSDE circuit where *M* = *N* = 8.

The performances of the 2-D networked resistive circuits were affected by *R_0_* (*R_0_* = *R_er_* = *R_ec_*), including the wire resistance and the contacted resistance caused by cables. As shown in Figure 1, the switch-on resistance of the *M*:1 multiplexers in the two-wire VF-NSDE circuit was also included in *R_er_* (*R_0_* = *R_er_*). Thus, the resistance of *R_0_* could be several hundred ohms. We investigated the effect of *R_0_*, including wire resistance and contacted resistance on the one-wire VF-NSDE circuit and the two-wire VF-NSDE circuit in NI Multisim. In simulations, we fixed some parameters, including the resistance value of the sampling resistor and all elements in the resistive sensor array at 10 kΩ, and *M* and *N* at 8, *R_0_* = *R_er_* = *R_ec_* in the sensor arrays varied synchronously with the same resistance value from 0.1 Ω–100 Ω. The simulation results of the two circuits in NI Multisim 12 were shown in Figure 2.

From Figure 2, with *R_0_* varied from 0.1 Ω to 100 Ω, *R_xy_* errors in the one-wire VF-NSDE circuit showed a large change (from 0.006% to 8.958%) with an obvious positive coefficient, while *R_xy_* errors in the two-wire VF-NSDE circuit showed a tiny change (from −0.003% to −0.002%). Thus, the two-wire VF-NSDE circuit has a better performance than the one-wire VF-NSDE circuit when *R_0_* is varied from 0.1 Ω to 100 Ω; the absolute *R_xy_* errors in the one-wire VF-NSDE circuit are small enough to be negligible (less than 0.087% for the one-wire VF-NSDE circuit) when *R_0_* is less than 1 Ω; the absolute *R_xy_* errors in the two-wire VF-NSDE circuit are small enough to be negligible (less than 0.003% for the two-wire VF-NSDE circuit) when *R_0_* is less than 100 Ω.

#### 3.1.2. Array Size Effect Simulation Experiment

**Figure 3:**
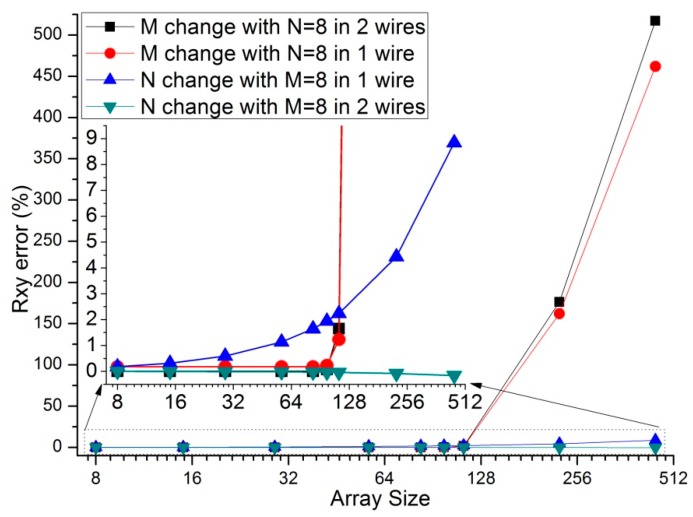
Array size effect on the *R_xy_* errors in the one-wire VF-NSDE circuit and the two-wire VF-NSDE circuit where *R_0_* = 2 Ω and *R_other_* = 10 kΩ.

Parameters of the array size, such as the row number (*M*) and the column number (*N*), were proved to have effects on the performance of the 2-D networked resistive sensor arrays [9,10,11,12,13,14,15,16,17]. We investigated the effect of *M* and *N* on the one-wire VF-NSDE circuit and the two-wire VF-NSDE circuit in NI Multisim. In simulations, we fixed some parameters including the resistance value of the sampling resistor and all elements in the resistive sensor array at 10 kΩ, *M* or *N* at 8, *R_0_* at 2 Ω, *N* or *M* was one number in (8, 15, 29, 57, 83, 98, 113, 225, and 449). The array size effect on the one-wire VF-NSDE circuit and the two-wire VF-NSDE circuit was simulated in NI Multisim and the results were shown in Figure 3.

From Figure 3, the absolute *R_xy_* errors in both circuits increased with the increase of the row number or the column number; with the increase of the column number, the *R_xy_* errors in the one-wire VF-NSDE circuit had a positive coefficient (from 0.178% to 8.85%) while the *R_xy_* errors in the two-wire VF-NSDE circuit had a negative coefficient (from −0.002% to −0.162%), and the absolute *R_xy_* errors in the two-wire VF-NSDE circuit had been reduced significantly comparing with the absolute *R_xy_* errors in the one-wire VF-NSDE circuit.

From Figure 3, with the increase of the row number, the *R_xy_* errors in the two-wire VF-NSDE circuit were similar to the *R_xy_* errors in the one-wire VF-NSDE circuit; with the row number changed in the range from 8 to 98, the *R_xy_* errors in both circuits changed small (from 0.178% to 0.231% for the one-wire VF-NSDE circuit, from −0.002% to 0.077% for the two-wire VF-NSDE circuit); but when the row number changed in the range from 113 to 449, the *R_xy_* errors in both circuits changed greatly (from 1.23% to 462% for the one-wire VF-NSDE circuit, from 1.65% to 517% for the two-wire VF-NSDE circuit). While both circuits had too many row lines, the hypothesis of the op-amps’ virtual short was not correct as every column-driving op-amp had a limited ability in driving current. Thus, in the two-wire VF-NSDE circuit, the influence of the array size on the *R_xy_* error has been decreased greatly; fewer row numbers and greater column numbers are preferred for good performance.

#### 3.1.3. The Adjacent Elements Effect Simulation Experiment

**Figure 4:**
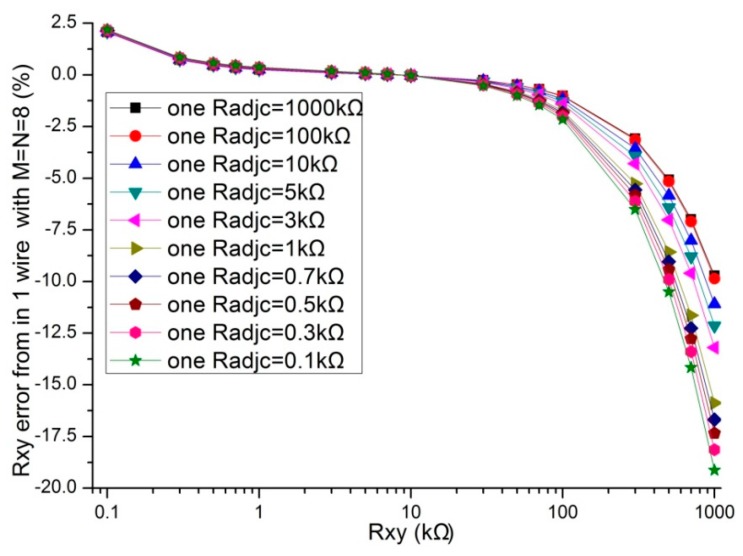
The *R_adjc_* effect on *R_xy_* errors in the one-wire VF-NSDE circuit.

**Figure 5:**
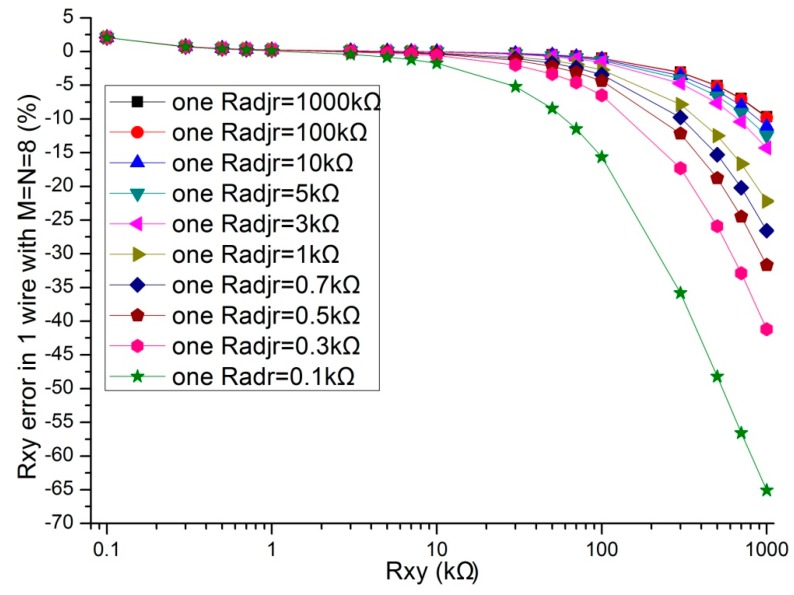
The *R_adjr_* effect on *R_xy_* errors in the one-wire VF-NSDE circuit.

**Figure 6:**
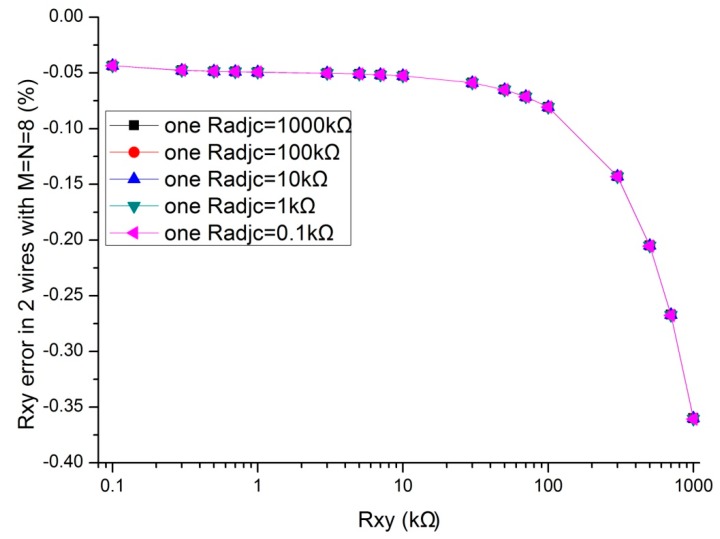
The *R_adjc_* effect on *R_xy_* errors in the two-wire VF-NSDE circuit.

**Figure 7:**
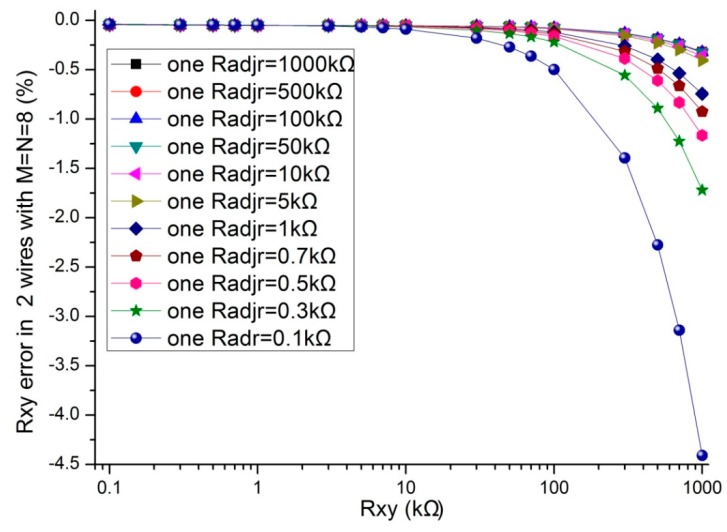
The *R_adjr_* effect on *R_xy_* errors in the two-wire VF-NSDE circuit.

In the literatures [9,10,11,12,13,14,15,16,17], the adjacent elements of all other elements played a significant role in affecting the measurement accuracy of the EBT. In simulations, we fixed some parameters, including the resistance value of non-adjacent elements and all other adjacent elements at 10 kΩ, *M* and *N* at 8, *R_0_* at 2 Ω, *R_s_* at 10 kΩ. The resistance value of the EBT and one adjacent element varied in the range from 0.1 kΩ to 1 MΩ. The adjacent element could be an adjacent row element (*R_adjr_*) or an adjacent column element (*R_adjc_*). The simulation results of the one-wire VF-NSDE circuit and the two-wire VF-NSDE circuit in NI Multisim are shown in Figure 4, Figure 5, Figure 6 and Figure 7.

From Figure 4, Figure 5, Figure 6 and Figure 7, the *R_xy_* errors in the EBT with a greater resistance value were more easily to be interfered by one *R_adjr_* or one *R_adjc_* with a smaller resistance value. In both circuits, the changes of the *R_xy_* errors for the change of one *R_adjr_* were larger than the changes of the *R_xy_* errors for the change of one *R_adjc_*. With one *R_adjr_* or one *R_adjc_* varied from 0.1 kΩ to 1 MΩ, the changes of the *R_xy_* errors (with *R_xy_* at 1 MΩ, from −9.72% to −19.1% for one *R_adjc_* and from −9.67% to −65.1% for one *R_adjr_*) in the one-wire VF-NSDE circuit were significant, while those (with *R_xy_* at 1 MΩ, from −0.290% to −0.289% for one *R_adjc_* and from −0.247% to −4.34% for one *R_adjr_*) in the two-wire VF-NSDE circuit were small. Thus, in the two-wire VF-NSDE circuit, the influence of the adjacent elements on the *R_xy_* error has been decreased greatly.

### 3.2. Test Experiments with the Two-Wire VF-NSDE Prototype Circuit

A VF-NSDE prototype circuit based on the two-wire voltage feedback method was designed. In the prototype circuit, OPA4209 and OPA2209 (from the datasheet the offset voltage, the bias current, the gain-bandwidth, and the gain are equal to ±150 μV, ±1 nA, 18 MHz, and 120 dB, respectively) were used as the op-amps, MAX4617 and MAX4619 (from the datasheet the on-resistance, the on-resistance match between channels, and the on-resistance flatness are equal to 8 Ω, 0.2 Ω, and 1 Ω, respectively) were used as the multiplex switches, and the resistance of the sampling resistor was 10 kΩ. In the prototype circuit, *M* and *N* were 16 and 8, respectively, and 48 lines were necessary for the prototype circuit. A cable with 50 core lines and its length of 400mm was used to connect the resistive sensor array modules with the circuit. The remaining two lines connected to ground were used as the shield lines. In the prototype circuit, VI was 3.30 V and a micro control unit (MCU) with an embedded 8-channel, 12-bit analog-to-digital converter (ADC) was used.

#### 3.2.1. The *R_xy_* Measurement Experiment with the Two-Wire VF-NSDE Prototype Circuit

**Figure 8:**
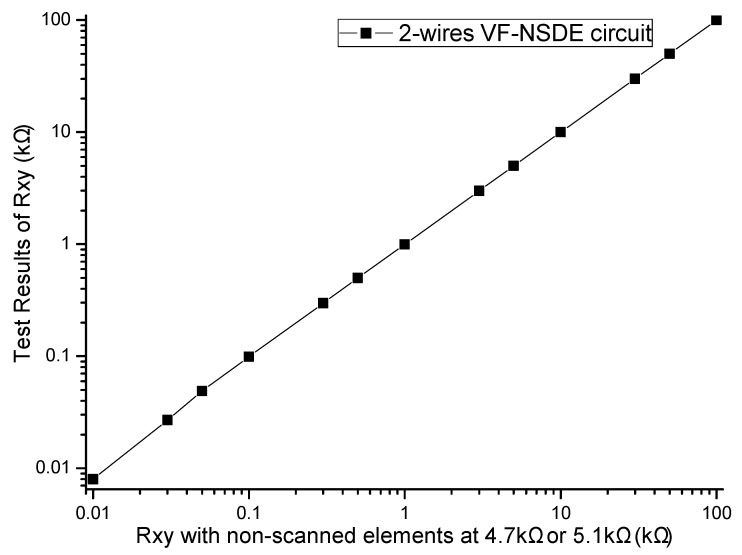
Results of the EBT varied from 10 Ω to 100 kΩ in the prototype circuit.

In the experiment, the EBT was replaced by a precision resistance box with its smallest step resistance value at 0.1 Ω, all non-scanned elements were precision resistors at 4.7 kΩ or 5.1 kΩ. The resistance value of the EBT was varied from 10 Ω to 100 kΩ, and the results of with the two-wire VF-NSDE prototype circuit were shown in Figure 8.

From the results in Figure 8, we found that the two-wire VF-NSDE circuit had a good performance in a wide range of the element being tested. Additionally, we found that the output of the circuit was unstable with the EBT of a larger resistance value (*R_xy_* > 50 kΩ). The reason could be the nonlinearity and the circuit noise of the voltage feedback circuit.

#### 3.2.2. The Adjacent Elements Effect Experiments with the Two-Wire VF-NSDE Prototype Circuit

**Figure 9:**
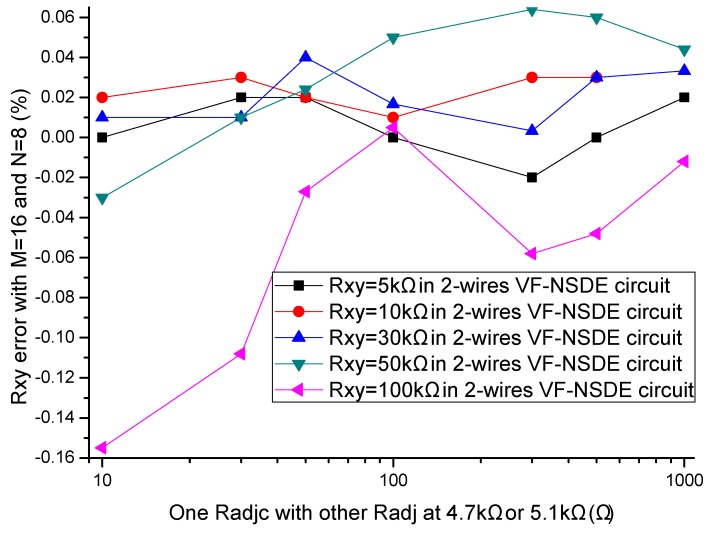
The *R_adjc_* effect on *R_xy_* errors in the two-wire VF-NSDE prototype circuit.

**Figure 10:**
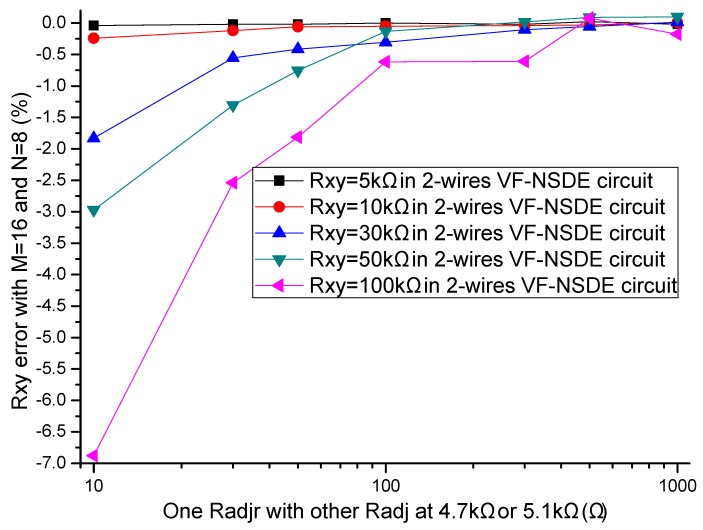
The *R_adjr_* effect on *R_xy_* errors in the two-wire VF-NSDE prototype circuit.

In the experiments, the EBT was replaced by a precision resistance box with its smallest step resistance value at 0.1 Ω, one adjacent element was also replaced by a precision resistance box, and the other non-scanned elements were precision resistors with each resistance value at 4.7 kΩ or 5.1 kΩ. The resistance value of the EBT varied in the range from 5 kΩ to 100 kΩ and the resistance value of one adjacent element varied in the range from 10 Ω to 1 kΩ. The adjacent element could be an adjacent row element (*R_adjr_*) or an adjacent column element (*R_adjc_*). The results of the two-wire VF-NSDE circuit were shown in Figure 9 and Figure 10. 

From the results in Figure 9 and Figure 10, in the two-wire VF-NSDE prototype circuit, we found that the adjacent row element with small resistance value (*R_adjr_* < 100 Ω) had a significant effect on the measurement error of the EBT. With a smaller resistance value of one row adjacent element, the effect was more significant. While the adjacent column element with small resistance value (*R_adjc_* < 100 Ω) had insignificant effect on the measurement error of the EBT. In simulation experiment results, similar variations were also found in Figure 6 and Figure 7. However, the variations in the two-wire VF-NSDE prototype circuit were smaller, which might be caused by the larger current driving feature in the op-amp of OPA4209. From the results in Figure 9 and Figure 10, we also found that the output of the circuit was unstable with the EBT of a larger resistance value (*R_xy_* > 50 kΩ).

### 3.3. Discussion

As shown in Figure 1, the one-wire VF-NSDE circuit had one voltage feedback op-amp, *N* 2:1 multiplexers, and *M* + *N* wires; the two-wire VF-NSDE circuit had one voltage feedback op-amp, *N* column driving op-amps, *N* 2:1 multiplexers, two *M*:1 multiplexers, and 2(*M* + *N*) wires. Thus, more components were used in the two-wire VF-NSDE circuit.

As shown in Figure 1, the two-wire VF-NSDE circuit had two *M*:1 multiplexers, which had the switch-on resistance of several hundred milliohms to several hundred ohms. The switch-on resistance of the *M*:1 multiplexers was included in *R_er_* (*R_0_* = *R_er_*). In practical circuits, the *M*:1 multiplexers with their switch-on resistances of small value, for example, 2 ohms, were preferred. From the results in Figure 2, we found that the multiplexers with the switch-on resistance of one hundred ohms could also be used in the circuits with the proposed method.

From the results in Figure 6, Figure 7, Figure 9 and Figure 10, with the change of one adjacent element, similar variations of the *R_xy_* error were found in the simulation circuit and the prototype circuit. However, the variations in the two-wire VF-NSDE prototype circuit were smaller, which might be caused by the larger current driving feature in the OPA4209 op-amp. Thus, a precision op-amp with large current driving features may better for a good performance of the two-wire voltage feedback circuit. From the results in Figure 2 and Figure 3, and Figure 6, Figure 7, Figure 8, Figure 9 and Figure 10, the good performance of the two-wire VF-NSDE circuit was verified with simulation experiments and practical circuit experiments. From the results in Figure 8, Figure 9 and Figure 10, we also found that the output of the circuit was unstable with the EBT with a larger resistance value (*R_xy_* > 50 kΩ). The reason could be the nonlinearity and the circuit noise of the voltage feedback circuit.

From the experiment results, the two-wire voltage feedback method was verified to be efficient in suppressing the VF-NSDE circuit’s crosstalk caused by the row wires and the column wires. The two-wire voltage feedback method could also be useful for depressing the cable crosstalk in other voltage feedback circuits such as the VF-NSE circuit, and the VF-NSSE circuit. It should be noted that all conclusions were correct under the assumption that the column driving op-amps had sufficient driving ability and the voltage feedback op-amp had very large input impedance on its non-inverting input.

From the results in Figure 3, Figure 6 and Figure 7, the two-wire VF-NSDE circuit failed to work normally with too large a row number, and its *R_xy_* error increased with the increase of the EBT’s resistance and the decrease of the adjacent element’s element. If the resistances of the adjacent elements in a resistive sensor array were too small, or the array size was too large for the column driving op-amps’ limited driving ability, *V_cy_* would be not equal to *V_I_* and the voltages on non-scanned column lines would not equal to *V_F_*. At the same time, if the voltage feedback op-amp did not have very large input impedance or the elements in the resistive sensor array had very large resistance values for the voltage feedback op-amp’s input impedance, *V_F_* would be not equal to *V_rx_*. Thus, the ideal work conditions would be destroyed for the two-wire VF-NSDE circuit and the *R_xy_* error would be significant.

## 4. Conclusions

Firstly, a two-wire VF-NSDE method of the 2-D networked resistive sensor array was proposed in this paper. Secondly, the formula was given for the equivalent resistance expression of the element being tested in the networked resistive sensor array by principle analysis. Thirdly, the effects of some parameters on the measurement accuracy of the EBT were simulated with the National Instrument Multisim 12, the parameters including wire resistances and contacted resistances of the long cables, the column number, the row number, and the adjacent elements of the 2-D resistive sensor array. Following this, a two-wire VF-NSDE prototype circuit was designed and its performance was tested by experiments. The experiment results show that the two-wire voltage feedback method is efficient in suppressing the crosstalk caused by the row wires and the column wires; in the 2-D networked resistive sensor array with the two-wire VF-NSDE circuit, the influence of the adjacent column elements and the adjacent row elements on the measurement error of the element being tested has been reduced greatly; fewer rows and more columns are preferred for good performance in accessing all elements individually in the 2-D resistive sensor array.
